# Anti-Microbial Potential of Nano-Emulsion form of Essential Oil Obtained from Aerial Parts of *Origanum Vulgare* L. as Food Additive

**DOI:** 10.34172/apb.2021.028

**Published:** 2020-02-23

**Authors:** Reza Enayatifard, Jafar Akbari, Amirhossein Babaei, Seyyed Sohrab Rostamkalaei, Seyyed Mohammad Hassan Hashemi, Emran Habibi

**Affiliations:** ^1^Department of Pharmaceutics, Faculty of Pharmacy, Mazandaran University of Medical Sciences, Sari, Iran.; ^2^Student Research Committee, Department of Pharmaceutics, Faculty of Pharmacy, Mazandaran University of Medical Sciences, Sari, Iran.; ^3^Department of Pharmacognosy and Biotechnology, Pharmaceutical Sciences Research Center, Hemoglobinopathy Institute, Faculty of Pharmacy, Mazandaran University of Medical Sciences, Sari, Iran.

**Keywords:** Anti-microbial, Nanoemulsion, Essential oil, *Origanum vulgare*, Food additive

## Abstract

***Purpose:*** Foodborne diseases are still a serious problem in public health and natural compounds are being widely considered for their potential industrial protective additive in food products. *Origanum vulgare* L. has been known as an antimicrobial effective herb. This present study was carried out to examine the antimicrobial effect of *O. vulgare* essential oil nanoemulsion in comparison with conventional emulsion.

***Methods:*** The essential oil was obtained by hydrodistillation, analyzed by GC-Mass and formulated as a nanoemulsion to improve water dispersion by high-energy emulsification method. The antimicrobial activity of the prepared formulation was assessed by measuring the minimum inhibitory concentration (MIC), minimum bactericidal/fungicidal concentration (MBC/MFC) and zone of inhibition against some main foodborne pathogen microorganisms.

***Results:*** The main component of the oregano essential oil was carvacrol (78%) and the selected nanoemulsion formulation demonstrated low polydispersity (0.11) and mean droplet (72.26 nm) and it was stable even after 30 days of storage. The nanoemulsion form displayed significant activity against the *Staphylococcus aureus*, *Candida albicans* and *Aspergillus niger* with inhibition zones ranging from 8.7–22.3 mm. The MIC of nanoemulsion against the tested bacteria was within the range of 0.156 to 0.312 (mg/mL) and against the tested fungi were in the range of 0.078 to 0.156 (mg/mL). The MBC/MFC of nanoemulsion against the tested microorganisms were in the range of 0.312 to 5 (mg/mL).

***Conclusion:*** The study’s results demonstrated the possibility of using the nanoemulsion form of oregano essential oil as a food additive to inhibit the growth of some foodborne microorganisms and extending the shelf life of food products.

## Introduction

Nowadays, microbial contamination of food is one of the major problems of the food industry and the general public health in the world. Foodborne disease for example diarrheal disease kills a lot of people in the world every year.^1^ Since long-term use of synthetic anti-microbial preservatives and food additives (like parabens) have adverse side effects and make changes to the natural form of foods, also the economic effects of corrupted foods and the consumer’s concerns over the safety of foods containing synthetic chemicals demonstrate the importance of using natural antimicrobial compounds or naturally derived compounds in food industries.^2-4^ If the level of synthetic antimicrobial compounds in foods is reduced, the need for other additives will be required to maintain the safety of food. One of the natural possibility is the use of essential oils obtained from plant materials as antimicrobial additives^5-7^ and in more recent studies examined for their effectiveness for food safety and preservation applications.^8-10^ Essential oils are complex blends of various different volatile and aromatic substances extracted from different plant organs that dissolve in low quantities in water and are used in the perfume and food industries as a fragrance and flavoring agent.^11^ Many essential oils have been displayed to possess strong antibacterial, antiviral, and antifungal effects.^1,12,13^

*Origanum vulgare* L., Lamiaceae family, also known as oregano is an annual, perennial and shrubby safe flavoring herb that traditionally used worldwide for its medicinal purposes (dental caries, indigestion, respiratory disorders, rheumatoid arthritis, and urinary tract disorders) at home remedy and distributed widely throughout Asia, particularly in Iran.^14^ Earlier studies have reported anti-mutagenic, anti-oxidant, anti-hyperglycemic, anti-fungal, anti-viral, anti-inflammatory and potent anti-bacterial effects of this plant.^15^ Also previous studies have shown that the *O. vulgare* essential oil has antioxidant capacity and anti-microbial effect which has been related to terpenoid and phenolic components such as thymol, carvacrol, γ-terpinene, p-cymene, sabinene, caryophyllene, germacrene, spathulenol.^16,17^ The low solubility of essential oils in water is a technological limitation in industry, so it needs new methods for formulation. Nanoemulsions as very small droplet-sized emulsions are new nanometric drug delivery systems that are used for bioactive components and because of their simplicity of formulation and acceptable functional properties like physicochemical, more stability and the potency of ameliorating biological activity of hydrophobic compounds (increasing the surface area and interactions between active ingredients with biological membranes), these are suitable for utilization in food products.^2,18^ New researches have demonstrated an improvement of the physical properties of essential oils-loaded nanoemulsions as to their similar typical emulsions and the use of nanosized delivery systems can potentially increase passive cell absorption mechanisms, thus reducing resistance to mass transfer and increasing antimicrobial action. Further, it has been also noted a higher antimicrobial activity in essential oils nanoemulsions.^19-21^ Prior to this, essential oil nanoemulsions were documented to be effective as antimicrobials; However, data on the advantages of using them against microorganisms in the food industry is currently limited. The purpose of the present study was outlined to produce stable nanoemulsion from *O. vulgare* and then examined its protective action against some selected food contaminant microorganisms.

## Materials and Methods

### Materials 

Nonionic surfactant (Tween 80, Tween 20, Span 80), DMSO, Mueller-Hinton agar, Tryptic Soy broth, Tryptic Soy agar, Sabouraud Dextrose agar were purchased from Merck Millipore (Darmstadt, Germany). *Staphylococcus aureus* (PTCC 1112), *Escherichia coli* (PTCC 1330), *Bacillus subtilis* (PTCC 1023), *Pseudomonas aeruginosa* (PTCC 1074), *Salmonella Typhi* (PTCC 1609), *Candida albicans* (PTCC 5027), *Aspergillus niger* (PTCC 5011) were purchased from Persian Type Culture Collection (PTCC), Tehran, Iran.

### Plant materials

The aerial parts of the cultivated *Origanum vulgare* L.subsp *. vulgare* (Lamiaceae) were collected in July 2016 from the Hezarjirib region of Mazandaran in Iran and dried at room temperature in the absence of sunlight. The collection was under specialist supervision and it was finally confirmed by senior plant taxonomist. The voucher specimens have been deposited at the Herbarium of the Department of Pharmacognosy (Voucher No: E1-36-491), Faculty of Pharmacy, Mazandaran University of Medical Sciences, Sari, Iran.

### Essential oil isolation

The essential oil of aerial parts of *O. vulgare* was obtained using hydro-distillation for 4 hours by British type Clevenger apparatus. The obtained essential oil was dewatered with anhydrous sodium sulfate and stored under dark conditions at 4°C in a well-closed vial prior to use.

### Gas chromatography/mass spectrometry analysis

Analyses by GC–MS were carried out by an Agilent 5975 GC-MSD system. HP 5MS equipped with a DB-1MS column (30×250 µm, 0.25 μm) were used with gas of helium as a mobile phase (40 mL/min). The temperature program was 50°C for 5 minutes, 5°C/min to 240°C, then 10°C/min to 290°C. The split ratio was 1:50 in this procedure. The injector temperature was modified to 290°C and mass spectra were recorded at 70 eV. The mass range was set up from 35 to 450 m/z. For the injection (split injector), 5 μL of essential oil was diluted in 500 μL of hexane, and 5 μL of this diluted solution was injected. Identification and quantity of *O. vulgare*’s volatile oil constituents were made on the basis of their retention times, the peak area of spectrograms and their mass spectra, which were confirmed by comparison with data from the Wiley mass spectral library.^22^

### Formulation preparation

All formulation produced were o/w. Emulsions with varying droplet sizes named E (conventional emulsion) and N (nanoemulsion that included nano-sized droplets) were generated to evaluate their antimicrobial properties. The E emulsion was derived by emulsifying the essential oil (1% w/v) with the water phase consisting of Tween 80 and homogenization was achieved with an IKA Mixing Overhead Stirrer, Eurostar (IKA, Werke, Germany) at 1000 rpm for 10 minutes and then decreased to 500 rpm for 10 minutes. The N emulsions were generated by high-speed homogenizer using fixed essential oil concentration (1% w/v), nonionic surfactant (Tween 80, Tween 20, Span 80) and deionized water. Water was added to the organic phase contained surfactant and oil in various surfactant to oil ratio (SOR) with different hydrophilic-lipophilic balance (HLB) and simply mixed using a high-speed homogenizer (Silent Crusher M, Heidolph, Germany) at 8000 rpm for 15 minutes. Then the prepared emulsion was subjected to sonication using an ultrasonic sonicator (HD 3200, Bandelin, Germany) operating at a constant frequency of 20 kHz and amplitude (AM) of 25% equipped with a high-grade titanium tip for 10 min. With an outer ice-water bath, the temperature of the nanoemulsions was kept below 15°C across processing. All prepared nanoemulsions were stored at 25°C and the size and polydispersity index (PDI) of the different formulations were analyzed and finally, the most suitable formulation from a series of preliminary experiments was selected for this study.

### Characterization of nanoemulsion

The droplet size and PDI of the nanoemulsion particles were analyzed by the photon correlation spectroscopy instrument (Zetasizer ZS, Malvern UK). Nanoemulsion was diluted with water (1:10) for measurement. Assessment were made in triplicate and the average droplet size was represented as the mean diameter.^23^

### Transmission electron microscopy (TEM)

Morphology of the nanoemulsion droplets was determined by TEM using a Philips EM 208 S instrument. One drop of nanoemulsion was placed on a copper grid then stained by phosphotungstic acid solution 2% for 1 minute. The grid was observed at an acceleration voltage of 100 kV.

### Time stability of nanoemulsions

The stability of nanoemulsions was assessed by analyzing the droplet size changes at room temperature during 30 days of storage.

### Microbial culture and preparation of inoculum suspensions 

To reviving freeze-dried microorganisms, the bacterial strains were cultured in Tryptic Soy agar plates for 24 hours at 35°C, *C. albicans* and *A. niger* in Sabouraud Dextrose agar plates at 25°C for 24-48 hours and 1 week respectively. Short term storage of each culture was stored at 4°C as working stocks. Before each experiment, fresh culture of each strain was prepared by streaking colonies from working stock on fresh plates to obtain cells in stationary growth phase (all strain cultured in the above-mentioned condition). After incubation, well-isolated colonies were removed with a sterile wire loop and suspended in sterile saline water to obtain a standardized suspension. The turbidity of the *C. albicans* and bacterial suspensions were adjusted to 0.5 McFarland standard (equivalent to (1-5) × 10^6^ CFU/mL and (1-2) × 10^8^ CFU/mL) respectively. *A. niger* spores were suspended in sterile normal saline containing 0.05% Tween 20 and the turbidity was measured at 530 nm and transmission was adjusted to 80%-82%, corresponding to (0.5-4.5) × 10^6^ CFU/mL.^24^

### Determination of minimum inhibitory concentration (MIC)

The MIC is the lowest antimicrobial agent concentration that inhibits the visible growth of the microorganism tested and measures a bacteriostatic effect of them without any data on the condition the microorganism population.^25^

The MIC of prepared formulation for the tested microorganisms was specified as the value where no microorganism growth was observed in comparison with the control tubes.

Macro dilution is one of the most basic antimicrobial reliable testing methods that involved a 2-fold serial dilution of the original antimicrobial agents tubes.^26^ Serial 2-fold dilutions (1/2, 1/4, 1/8, 1/16, 1/32, 1/64, 1/128, 1/256) of 1% (w/v) nanoemulsion and emulsion, ranging from 5 to 0.039 (mg/mL), were made in sterile tube contained 5 mL Tryptic Soy broth. Finally, standard inoculum suspensions were added to each tube as the final concentration of the bacteria and *C. albicans* were 5 × 10^5^, (0.5-2.5) × 10^3^ and for *A. niger* was (0.4-5) × 10^4^ CFU/mL. After mixing, tubes are incubated at 35°C for 20-48 hours.^26^ Appropriate controls, including tubes with microorganism but no nanoemulsion/emulsion and tubes with nanoemulsion/emulsion dilutions but no microorganism, were simultaneously investigated. The examinations were performed in triplicate.

### Determination of minimum bactericidal/fungicidal concentration (MBC/MFC)

MBC/MFC was determined after assessing the MIC of the prepared formulation by adding 100 μl from tubes that had no visible microorganism growth to a suitable medium. Tryptic soy agar and Sabouraud Dextrose agar were chosen for bacterial and fungi strains respectively. The petri dishes were then incubated for 24 hours at 35°C, (except *A. niger* 48 hours). The MBC/MFC value is characterized as the lowest concentration of antimicrobial agent that kills >99.9% of the primary colony where no visible growth of the microorganism was observed. All assays were repeated three times.^27^

### Agar well diffusion assay

The antibacterial activity of emulsion and nanoemulsion of *O. vulgare*’s essential oil was carried out by agar well diffusion method.^28^ The standard inoculum suspensions of each bacteria and fungi were streaked on the Muller Hinton agar and Sabouraud Dextrose agar Petri dishes using sterile swap, respectively. Later agar media plates were punctured in 0.6 cm diameter by a sterile borer and each well separately loaded with 100 µL of different concentrations of nanoemulsion and emulsion ranging from 10 to 1.25 (mg/mL). The zone diameters of inhibition in the petri dishes were recorded after 16-18 hours of incubation for bacteria, 20-24 hours for *C. albicans* and 2-3 days for *A. niger* at 35°C.^26^ Amikacin, gentamycin, vancomycin, and amphotericin B were used as a positive control.

### Statistical analysis

All data were reported as mean ± standard deviation (SD), resulting from the assays. The statistical significance of the variations was investigated by SPSS software Version 16 using one-way variance analysis (ANOVA) and statistical significance was accepted at a level of *P <*0.05.

## Results and Discussion

### Essential oil composition

The yield of essential oil from *O. vulgare* was 0.1 % (w/w), respectively. The main constituents identified in *O. vulgare *’s essential oil are listed in Table 1. Eleven components involving 97.99% of total concentration of the essential oil. Carvacrol (78.46%), thymol (7.88%), benzene (7.03%) and gamma-terpinene (4.38%) were the main compounds identified in *O. vulgare * subsp *. vulgare* collected from the Hezarjirib region of Mazandaran in Iran.

**Table 1:** *Origanum vulgare*’s essential oil composition

**Components**	**Retention time (R**_t_ **)**	**Relative area (%)**
*β*-Pinene	9.184	0.05
p-Menthane	9.664	0.02
Benzene	11.026	7.03
1,8-Cineol	11.186	0.06
o-Cymene	11.450	0.02
Gamma-terpinene	12.199	4.38
Trans-beta-ocimene	11.839	0.03
Sabinene	12.623	0.02
Beta-phellandrene	12.668	0.04
Thymol	19.483	7.88
Carvacrol	20.279	78.46

### Formulation preparation

Different formulations were tested to providing the most stable nanoemulsion form of *O. vulgare*’s essential oil (1%) as antimicrobial purpose. Formulation, HLB, mean droplet size, and PDI are reported in Table 2. The mixed surfactants HLB values are a critical factor in emulsion droplet formation. The lipophilic surfactants have a greater affinity than the hydrophilic surfactant to the dispersed droplets in the emulsion during the forming of an o/w emulsion.^29^ To maintain the balance of the water and oil phases, proper HLB values are needed. During emulsification, the newly formed droplets are stabilized and their droplet size is maintained at an optimum HLB value. With an HLB value of 7.87, the droplet size was 366 nm, and by rising the HLB value to 15 the size of the droplet was 72 nm. The ratio of surfactant to oil is a significant factor that affects droplet size. Increasing the emulsifier amount will result in increased adsorption around the oil-water droplet interface and decreased process interface tension to form nanoemulsion with smaller droplets.^30^ The interface tension which is directly relevant to the SOR should be set to a suitable amount for the optimal droplet size distribution. At different SOR, nanoemulsion was prepared separately, and PDI of formulation observed by dynamic light scattering technique are shown in Table 2. At SOR 1:1, the PDI was obvious as multi branches, one branch with a significant amount of droplets in the range of nanometers, and some other branch with a range of micrometers, indicating that due to the emulsifier deficiency, the oil cannot be fully emulsified. As the SOR increased, the particle size distribution decreased significantly (*P <*0.05). More specifically, the formulations differed in the choice of the emulsifiers and surfactants, which ranged from Tween 20, Tween 80 and Span 80. The results showed that the lowest particle size and the most suitable PDI were obtained with Tween 80 as surfactant and SOR 3:1. However, the use of ultrasonication significantly reduced the particle size (*P <*0.05). But without this equipment and only with the aid of highspeed homogenizer nanometer particles were obtained and finally, formulation F2 was selected.

**Table 2:** Composition (% w/v ) of *Origanum vulgare* essential oil nanoemulsion

**Formulations**	**Essential oil**	**Tween 80**	**Tween 20**	**Span 80**	**Deionized water (ml)**	**Sonication**	**SOR**	**HLB**	**Mean diameter (nm)**	**PDI [-]**
F1	1	1	-	-	Up to 100		1	15	42.56±4.15	0.473±0.032
F2	1	3	-	-	Up to 100		3	15	72.26±3.62	0.113±0.005
F3	1	4	-	-	Up to 100		4	15	78.96±3.65	0.106±0.005
F4	1	3	-	-	Up to 100	10 min AM 25%	3	15	48.76±2.34	0.19±0.01
F5	1	3	-	-	Up to 100	10 min AM 50%	3	15	52.5±2.75	0.196±0.015
F6	1	-	1	-	Up to 100		1	16.7	81.63±3.32	0.443±0.051
F7	1	-	2	-	Up to 100		2	16.7	77.06±3.70	0.326±0.033
F8	1	-	4	-	Up to 100		4	16.7	207.43±6.41	0.156±0.015
F9	1	1	-	2	Up to 100		3	7.87	366.23±13.25	0.936±0.056
F10	1	1.5	-	1.5	Up to 100		3	9.65	343.06±12.15	0.9±0.04

### Transmission electron microscopy 

**Figure 1:**
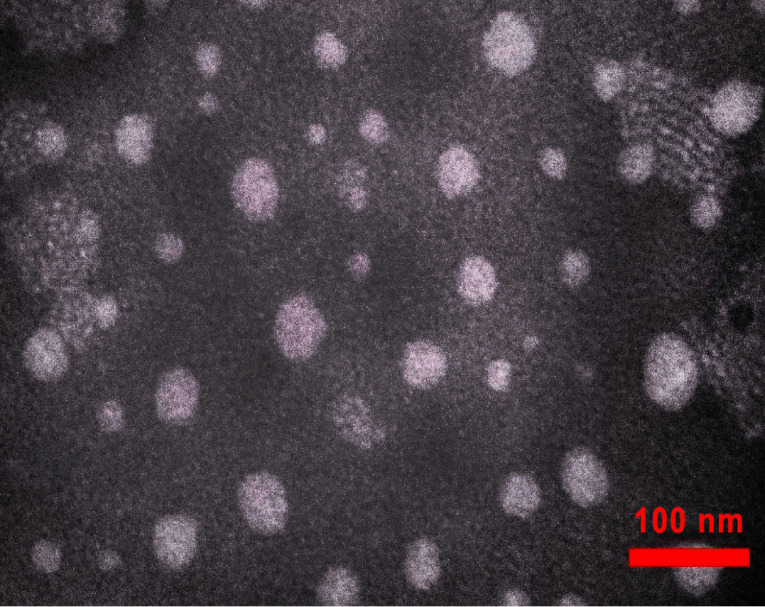
Transmission electron micrograph of essential oil nanoemulsion (F2).

TEM characterization of essential oil nanoemulsion showed the spherical and homogeneous shape of droplets (Figure 1). TEM micrograph also confirmed the results obtained from droplet size analysis using the dynamic light scattering with diameter <80 nm.

### Time stability of nanoemulsions

**Figure 2:**
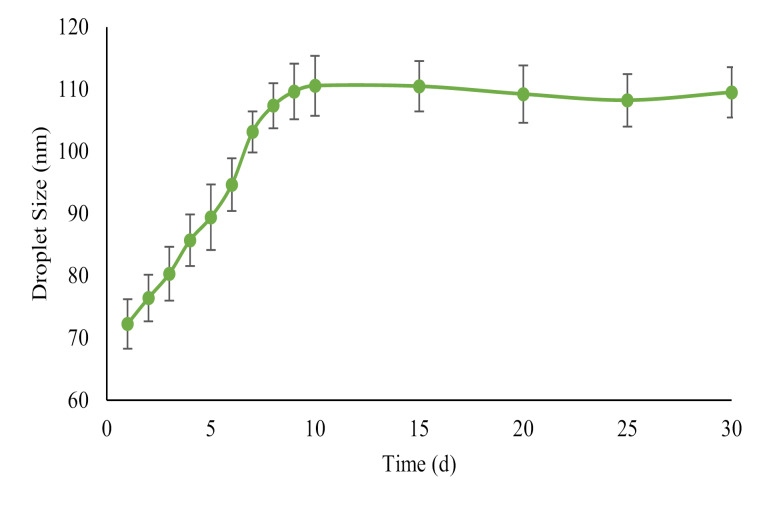
Stability of nanoemulsion (F2) stored at room temperature.

Changes in droplet size are shown in Figure 2 as a function of the storage time of nanoemulsions stored at room temperature. The droplet size raised very rapidly at room temperature during the initial 7 days of observation. The growth rate slowed down after 7 days, but the size of the nanoemulsions was still within appropriate range. The droplet size increased from 72 nm on production day to 109 nm on storage day 30.

### MIC, MBC and MFC of nanoemulsion and emulsion 

In the present study, the MIC and MBC/MFC of emulsion and nanoemulsion of *O. vulgare*’s essential oil were evaluated against several microorganisms known as foodborne pathogens. Generally, the MIC and MBC/MFC values of emulsion and nanoemulsion for the tested bacteria and fungi were found to be similar. The MIC of these agents against the tested bacteria were in the range of 0.156 to 0.312 (mg/mL) and against the tested fungi were in the range of 0.078 to 0.156 (mg/mL). The MBC/MFC of both emulsion and nanoemulsion against the tested microorganisms were in the range of 0.312 to 5 (mg/mL) and both of them had no effect on *B. subtilis*, *P. aeruginosa,* and *A. niger*. All of these findings are summarized in Table 3.

**Table 3:** MIC, MBC and MFC values (mg/mL) of nanoemulsion (N) and emulsion (E) forms of *O. vulgare*’sessential oil on selected microorganisms determined by macro dilution method

	***S. aureus***		***E. coli***		***B. subtilis***		***P. aeruginosa***		***S. typhi***		***C. albicans***		***A. *** ***niger***	
	**N**	**E**	**N**	**E**	**N**	**E**	**N**	**E**	**N**	**E**	**N**	**E**	**N**	**E**
MIC	0.312	0.312	0.312	0.312	0.312	0.312	-	-	0.156	0.156	0.156	0.156	0.078	0.078
MBC, MCF	5	5	5	5	-	-	-	-	2.5	2.5	0.312	0.312	-	-

### Agar well diffusion assay

**Figure 3:**
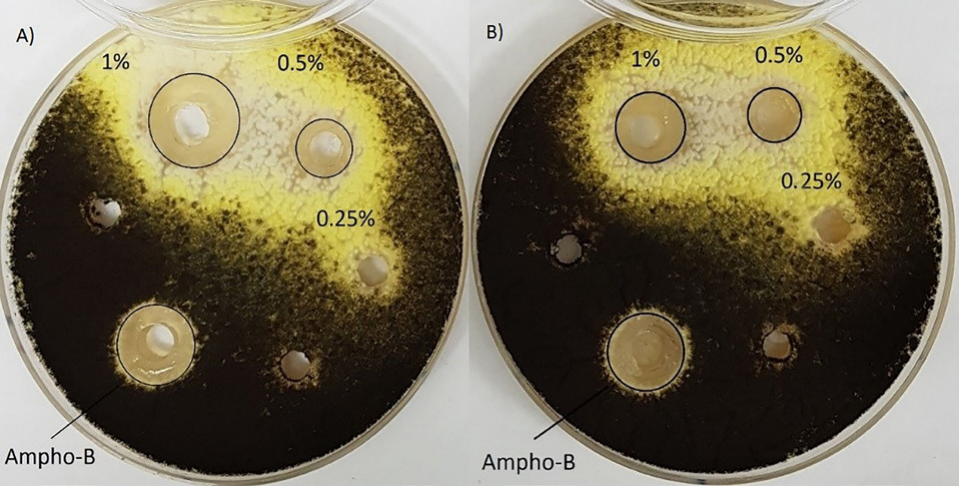
Zone of inhibition on A. niger by agar well diffusion. A) Nanoemulsion B) Emulsion.

**Figure 4:**
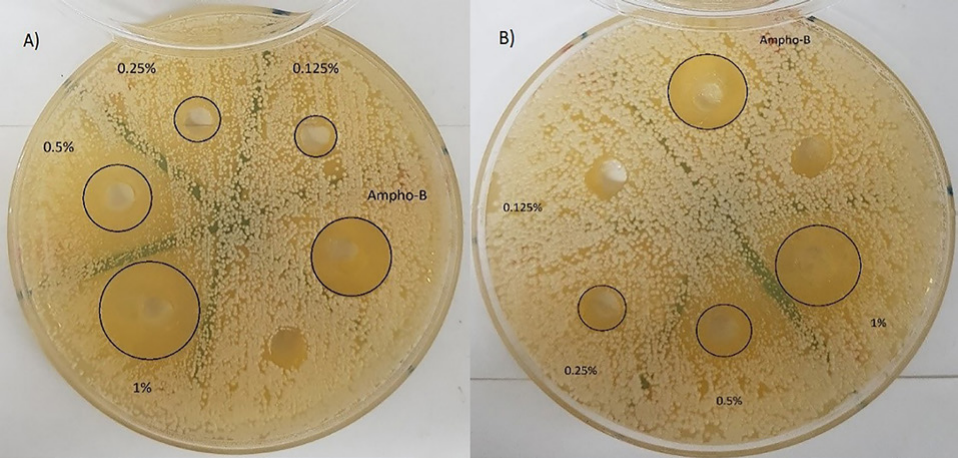
Zone of inhibition on C. albicans by agar well diffusion. A) Nanoemulsion B) Emulsion.

**Figure 5:**
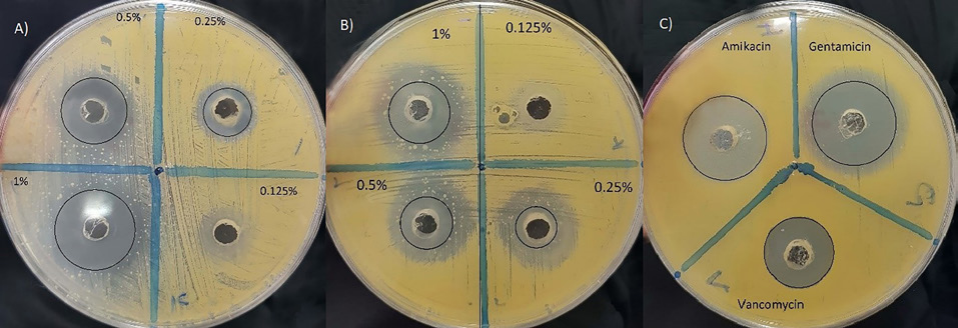
Zone of inhibition on S. aureus by agar well diffusion A) Nanoemulsion B) Emulsion C) Antibiotics as control.

The antimicrobial activity of the nanoemulsion of *O. vulgare*’s essential oil in different concentration (1.25-10 mg/mL) was evaluated by measuring the diameter of the inhibition zone using an agar well diffusion method shown in Table 4. It is clear that the emulsion and nanoemulsion form demonstrated significant activity against the *S. aureus*, *C. albicans* and *A. niger* (Figures 3-5) with inhibition zones ranging from 8.7–22.3mm, however, this agent had no inhibition effect on *E. coli*, * P. aeruginosa, B. subtilis*, and *S. typhi*. Control treatments (amikacin, gentamycin, amphotericin B and vancomycin) showed an inhibitory effect on tested microorganisms by inhibition zones ranging from 15.3–30.3 mm.

**Table 4:** Means of the inhibition diameters (mm) of nanoemulsion (N) and emulsion (E) forms of *O. vulgare*’sessential oil on selected microorganisms determined by agar well diffusion compared to standard drugs

**Concentration**	***S. aureus***		***E. coli***		***B. subtilis***		***P. aeruginosa***		***S. typhi***		***C. albicans***		***A. *** ***niger***	
	**N**	**E**	**N**	**E**	**N**	**E**	**N**	**E**	**N**	**E**	**N**	**E**	**N**	**E**
1.25 mg/mL	-	-	-	-	-	-	-	-	-	-	8.7±0.1	-	-	-
2.5 mg/mL	13±0.3	11.2±0.2	-	-	-	-	-	-	-	-	9.5±0.2	9.3±0.2	-	-
5 mg/mL	18.6±0.7	14.6±0.8	-	-	-	-	-	-	-	-	14.2±0.2	10.8±0.3	11.2±0.4	10.3±0.3
10 mg/mL	22.3±0.8	16.8±0.6	-	-	-	-	-	-	-	-	20.6±0.9	16.5±0.8	18±0.5	13.4±0.4
Amikacin (30 µg/mL)	23.1±0.7		23±0.6		30.3±0.5		19.4±0.6		24.2±0.6		-		-	
Gentamycin (10 µg/mL)	23.4±0.5		24.2±0.7		29.3±0.7		16.5±0.8		27.2±0.7		-		-	
Vancomycin (10 µg/mL)	20.1±0.4		-		26.9±0.7		-		-		-		-	
Amphotericin B (100 µg/mL)	-		-		-		-		-		15.9±0.3		15.3±02	

Plant essential oils have been observed as useful sources of antimicrobial agents and can be an appropriate antibacterial preservative at a concentration which does not turn out unfavorable changes in the flavor of food products but a few preservatives containing essential oils are already commercially available. *O. vulgare* is commonly recognized as having medicinal properties and its essential oil used in the food, agricultural, pharmaceutical and cosmetic industries and has shown antimicrobial activity against microorganisms.^1^ Essential oils, however, have low water solubility and this is a limiting factor for their usage as antimicrobial agents. A nanoemulsion of *O. vulgare* essential oil can solve the problem of low water solubility, so the purpose of this study was to promote and evaluate nanoemulsion formulations of oregano essential oil for its antimicrobial activity in comparison with emulsion form and standard amikacin, gentamycin, vancomycin, amphotericin B antibiotics (positive control).

There is currently a new trend in the use of natural plant substances as well as essential oils as natural antimicrobial agents to combat microbial spoilage with nanoformulations. Therefore, novel approaches are urgently needed to develop new green antimicrobial agents based on nanoformulations of essential oils as an alternative to synthetic anti-microbial preservatives for spoilage prevention. A silver nanoparticle from *O. vulgare*’s aqueous leaf extract has been green synthesized by reducing the solution of silver nitrate and this silver nanoparticles showed effective antimicrobial activity against some human pathogenic bacterial strains (namely: *A. hydrophila*, *Bacillus spp*., *E. coli*, *Salmonella* spp., *S. paratyphi*, *Klebsiella spp*., *S. dysenteriae*, *S. sonnei*) by agar well diffusion method compared to standard reference drug (chloramphenicol). The enhancing effect of bioactive compounds such as thymol and carvacrol was reported.^15^ Phenolic compounds like thymol, eugenol, and carvacrol compare to other constituents in the essential oils possess the noticeable antimicrobial activity^31,32^ and this effect is probably due to the interaction of the hydroxyl group of their molecules with the cell membrane and likely acts as donor of proton, that is a carrier of protons across the lipid bilayers, therefore causing the waste of the proton movement energy and finally causing leakage of cellular substances like potassium ions, increase in concentration of the saturated fatty acids and change phospholipids profiles membrane and causing membrane structural alterations, decrease of intracellular ATP as well as the increase of extracellular ATP.^19,33^ Baydar et al. reported the possibility of using the essential oils of the wild oregano (*Origanum minutiflorum*), oregano (*Origanum onites*) as natural food preservatives to preventing the growth of foodborne pathogens at 2% concentration and extending the shelf life of processed foods, due to the presence of the high amount phenolic compounds like carvacrol (about 80%) and to the hydrocarbons c-terpinene and p-cymene that possess strong antimicrobial properties.^34^ Analysis of essential oil composition of *O. vulgare subsp*. *glandulosum* (Desf.) and antibacterial activity against *S. aureus*, *B. subtilis*, *E. coli*, *S. typhimurium*, *P. aeruginosa* were investigated and the results closed to our study, high level of carvacrol about 80% and the inhibition zones and MIC values reported in the range of 9–36 mm and 125–600 μg/mL, respectively.^35^ Also, antimicrobial activity of *O. vulgare*’s essential oil was evaluated up to 72 h against foodborne pathogens (*S. aureus*, *L. monocytogenes*, *S. enteritidis*, *C. jejuni*) through disk diffusion and determination of MIC, that showed the 0.5% concentration of the essential oil of *O. vulgare* could be used as food additives without considerably making different the flavor of food and be able to act as bacteriostatic and bactericidal against high and low pathogen concentrations.^31^ In a previous study, *Origanum compactum*’s essential oil (2%) containing phenolic compounds like thymol and eugenol has been showed antimicrobial effect on *E. coli* and *B. subtilis* by inducing cell lysis and stimulating damage in the bacterial envelope, which plays a fundamental role in the life of bacteria. The results for *E. coli* were similar to those for polymyxin B and scanning electronic microscope observations revealed that both cell wall and membrane of the treated bacteria were significantly damaged^36^ however, in our study *O. vulgare* essential oil emulsion and nanoemulsion form in concentration up to 1 % showed no inhibition growth of *E. coli* and *B. subtilis*. Similarly, a nanoemulsion of thyme oil (*Thymus vulgaris* L.) prepared containing Tween 80 (3% v/v), Alginate dispersions and glycerol in a high-speed blender at room temperature for enhancing encapsulation of essential oil to obtaining functional edible film that exhibited strong inhibitory effect against inoculated *E. coli* because of high content of thymol in it. Thymol molecules can attach to the membrane proteins of microorganisms by hydrophobic interactions, thereby altering the permeability of the membrane, which may help to protect and preserve food products.^18^ As mentioned in the results, the nanoemulsion form of *O. vulgare*’s essential oil showed a specific effect on the fungi that confirms the presence of high content of phenolic compounds in it (Figures 3 and 4).

## Conclusion

In conclusion, design and suggestion of an appropriate emulsifier in the final formulation of the nanoemulsion form of *O. vulgare* essential oil (1%) exhibited significant activity against the *S. aureus*, *C. albicans,* and *A. niger* and it could be new antimicrobial agents with great potential usage in food industries as food preservatives. In fact, it is probably a delivery system capable of ensuring a lower concentration of antimicrobial compounds such as essential oils in the aqueous phase.

## Ethical Issues

Not applicable.

## Conflict of Interest

The authors declare no conflict of interest.

## Acknowledgments

This research was supported by grants from the Mazandaran University of Medical Sciences, Sari, Iran (no. 2219).
